# Case Report: Risk Factors Associated with Mortality in Adults with *Burkholderia Pseudomallei* Bacteremia: A Retrospective Case Series of Melioidosis in Cambodia

**DOI:** 10.4269/ajtmh.23-0369

**Published:** 2024-06-18

**Authors:** Andrea R. Pacheco, Sophana Chea, Helena Saunders, Sokna Ly, Ratanak Sath, Rathna Tim, Sreyngim Lay, Mengheng Oum, Gechlang Tang, Lyhourng Long, Sovann Ly, Heng Seng, Sidonn Krang, Chhouv Chhuon, Vantha Te, Sosorphea Seang, Chanthap Lon, Christina Yek, Jessica E. Manning

**Affiliations:** ^1^ International Center of Excellence in Research, National Institute of Allergy and Infectious Diseases, Phnom Penh, Cambodia;; ^2^ Department of Psychology, Colorado College, Colorado Springs, Colorado;; ^3^ National Center of Parasitology, Entomology, and Malaria Control, Phnom Penh, Cambodia;; ^4^ Communicable Disease Control Department, Ministry of Health, Phnom Penh, Cambodia;; ^5^ Takeo Provincial Referral Hospital, Takeo, Cambodia;; ^6^ Department of Critical Care Medicine, National Institutes of Health Clinical Center, Bethesda, Maryland;; ^7^ Laboratory of Malaria and Vector Research, National Institute of Allergy and Infectious Diseases, Rockville, Maryland

## Abstract

In resource-scarce settings, melioidosis is associated with up to 80% mortality. Studies of melioidosis in Cambodia report primarily on pediatric populations with localized infection; however, literature describing Cambodian adults with severe melioidosis is lacking. We present a case series of 35 adults with sequence-confirmed *Burkholderia pseudomallei* bacteremia presenting to a provincial referral hospital in rural Cambodia. More than 90% of the patients had diabetes, an important risk factor for developing melioidosis. Inappropriate antimicrobial therapy was significantly associated with lower odds of survival. Improved diagnostic testing and greater access to first-line antibiotics for acute melioidosis treatment present potential targets for intervention to reduce mortality associated with this disease in resource-limited settings.

## INTRODUCTION

Melioidosis, caused by the gram-negative environmental bacterium *Burkholderia pseudomallei*, is responsible for an estimated 4.64 million disability-adjusted life-years annually and poses a growing global threat.^1^ Sixty countries have confirmed endemicity, but the true burden may be underappreciated; crude models project an additional 22 countries where melioidosis has not yet been reported.^2^^,^^3^ In nonendemic areas, infected travelers or wildlife import the bacteria.^4^ Mortality rates range from 6% to 80%, with worse outcomes in resource-scarce settings.^3^^–^^6^ High-risk populations include adults with diabetes, chronic lung or renal disease, excessive alcohol use, and high occupational exposure (e.g., rice farmers).^4^^,^^5^^,^^7^

Despite recent efforts to recognize melioidosis as a neglected tropical disease,^1^ *B. pseudomallei* has received little attention outside the hyperendemic regions of Southeast Asia and northern Australia. Clinical data from melioidosis patients derive mostly from Australia, where timely diagnosis and appropriate treatment have brought mortality rates below 10%.^5^ In contrast, recent studies from Southeast Asia have estimated 60% mortality.^3^ In Cambodia, clinical appreciation has been relatively recent, and diagnostic capacity remains scattered.^8^ Published Cambodian reports have been largely limited to pediatric patients, reflecting overrepresentation from select pediatric hospitals with well-established microbiology laboratories.^4^^,^^6^^–^^10^ One small case series of seven adult patients reported mortality surpassing 50%.^11^ Rates of appropriate antibiotic treatment of melioidosis in Cambodia range from 33% to 66%, as hospital supplies are often insufficient or, in the case of carbapenems, nonexistent.^6^^–^^11^

Here, we describe clinical presentations and outcomes of systemic melioidosis in an adult cohort in rural Cambodia. Understanding key features leading to poor outcomes will add to improved management of *B. pseudomallei* bacteremia in the future.

## MATERIALS AND METHODS

The study protocol was approved by the Cambodian National Ethics Committee for Health Research as part of the antimicrobial resistance surveillance network. Cases were identified from patients presenting to Takeo Provincial Referral Hospital, a 250-bed government hospital serving approximately 900,000 people in southern Cambodia.^11^ Cases were defined as growth of *B. pseudomallei* from blood cultures drawn at the hospital and analyzed by the hospital’s microbiology laboratory between January 1, 2021 and June 15, 2022.

At the Takeo Hospital microbiology laboratory, blood cultures were processed in nonautomated systems. Laboratory technicians visually checked cultures for signs of bacterial growth every 24 hours, then performed Gram stain on positive cultures. If organisms morphologically suggestive of *B. pseudomallei* were seen, samples were directly plated using the three-antibiotic disk method for rapid identification.^8^ All other positive cultures were subcultured on sheep blood and/or MacConkey agar before further biochemical and antibiotic susceptibility testing for isolate differentiation and characterization.

Once *B. pseudomallei* infection was confirmed, deidentified patient chart data were entered into a standardized case report form, with primary site of infection assigned based on presenting symptoms, discharge diagnosis, and available imaging and comorbid conditions (e.g., chronic alcohol use, diabetes) obtained from physician documentation. Ceftazidime and carbapenems were considered appropriate antibiotics for acute treatment.^5^ Isolate identification was confirmed by sequencing at the National Institute for Allergy and Infectious Diseases/International Center of Excellence in Research in Phnom Penh using methods previously described.^12^

The primary outcome was in-hospital mortality (“death”) or discharge to hospice. Patients discharged with “no improvement” or “hopeless” documented in notes were classified as discharged to hospice given the local cultural tradition of dying at home, particularly where in-hospital care is unaffordable.^7^ Covariates were demographics (age, sex), comorbid diseases (diabetes, chronic alcohol use), infection site, and treatment details (time to presentation, time to positive culture, receipt of appropriate antibiotic therapy).

Normally and non-normally distributed data were reported using mean (SD) or median (interquartile range [IQR]), respectively. Univariate analyses were performed using Fisher’s exact test for categorical variables and *t*-test or Wilcoxon rank-sum/matched-pairs signed-rank tests for continuous variables with parametric and nonparametric distributions, respectively. Analyses were performed using GraphPad Prism v. 9.2.0 (332) (LaJolla, CA).

## RESULTS

### Patient population.

Of 178 blood cultures collected during the study period, 82 (46%) were Enterobacterales and 55 (31%) were initially identified as *B. pseudomallei* (Supplemental Table 1). Sequencing revealed that one isolate was misidentified *Klebsiella pneumoniae* and another *Achromobacter xylosoxidans*. Antimicrobial susceptibility testing revealed sensitivity to ceftazidime, amoxicillin-clavulanic acid, meropenem, and trimethoprim/sulfamethoxazole and resistance to gentamicin in all isolates.

Of the 53 sequencing-confirmed cases of *B. pseudomallei*, 35 had available patient records. Most patients were male (26/35), and the mean (SD) age was 56.1 (13.3) years (Table 1). Almost all patients (32/35) had diabetes. Twenty-three percent of patients (8/35) reported chronic alcohol use. Most cases (69%; 24/35) presented with primary pneumonia, 14% (5/35) with skin or soft-tissue infection, 14% (5/35) with intraabdominal infection, and 3% (1/35) with urinary tract infection. Symptom duration before admission was 4 days (IQR: 2.0–10.0), ranging from 1 day of fever and dyspnea for pneumonia to 90 days of epigastric pain for visceral abscesses. Eleven percent of patients initially sought treatment at an ancillary facility, of whom 27% (3/11) survived compared with 54% (13/24) of patients presenting initially to the referral hospital.

**Table 1:** Demographics, clinical features, and outcomes of 35 patients with bacteremic melioidosis

Variables	All Patients (*N* = 35) *n* (%)	Improved (*n* = 16) *n* (%)	Died or Discharged to Hospice (*n* = 19) *n* (%)	*P*-Value and OR (95% CI)
Demographics				
Age, Mean (SD)	56.1 (13.3)	56.2 (15.4)	55.9 (11.8)	*P* = 0.7746
Sex				
Male	26 (74)	11 (69)	15 (79)	–
Female	9 (26)	5 (31)	4 (21)	0.6 (0.2–2.8)
				*P* = 0.7003
Diabetes*	32 (91)	16 (100)	16 (84)	–
Hypoglycemic Use in Diabetic Patients (*N* = 31)^†^				
Oral and/or Insulin	25 (78)	15 (94)	10 (63)	–
None	6 (19)	1 (6)	5 (31)	7.5 (1.0–93.2)
				*P* = 0.0829
Chronic Alcohol Use*				
No	27 (77)	13 (81)	14 (74)	–
Yes	8 (23)	3 (19)	5 (26)	1.5 (0.3–6.7)
				*P* = 0.7003
First Treated at Ancillary Facility				
No	24 (69)	13 (81)	11 (58)	–
Yes	11 (31)	3 (19)	8 (16)	3.2 (0.7–12.7)
				*P* = 0.1667
Clinical Presentation				
Days of Symptom Duration before Admission, Median (IQR)	4.0 (2.0–10.0)	8.0 (3.8–11.3)	2.5 (2.0–10.0)	***P* = 0.0379**
Primary Site of Infection				
Respiratory	24 (69)	9 (56)	15 (79)	–
Skin/Soft Tissue	5 (14)	2 (13)	3 (16)	0.9 (0.2–5.8)
				*P* >0.9999
Intraabdominal	5 (14)	5 (31)	0	–
Urinary	1 (3)	0	1 (5)	–
Clinical Course				
Days Hospitalized, Median (IQR)	4.0 (2.0–16.0)	17.5 (13.8–21.3)	2.0 (1.0–3.5)	***P* <0.0001**
Days from Admission to Positive Culture, Median (IQR)	4.0 (2.5–6.0)	4.5 (3.0–5.3)	3.0 (2.0–6.0)	*P* = 0.5791
Appropriate Antibiotics Received^‡^^§^				
Ever	13 (37)	11 (69)	2 (11)	–
Never	18 (51)	3 (19)	15 (79)	**27.5 (4.1–148.2)**
				***P* = 0.0003**

IQR = interquartile range; OR = odds ratio. Statistically significant (*P* <0.05) differences (quantitative data) and ORs (qualitative data) are shown in bold.

* Comorbid conditions were obtained from physician documentation.

^†^ Oral hypoglycemic use was typically metformin, but four patients received glyburide alone or in combination with metformin; hypoglycemic data were incomplete for one patient (died/discharged to hospice) and excluded for three nondiabetic patients.

^‡^ For acute treatment, appropriate antibiotics included ceftazidime and carbapenems.

^§^ Medication data were incomplete for four patients (two improved; two died/discharged to hospice).

### Factors impacting mortality.

There were 10 deaths and nine discharges to hospice, accounting for an overall likely case-fatality rate of 54%. Deaths and discharges occurred a median of 7.0 (2.5–17.5) days after symptom onset and 2.0 (1.0–3.5) days after presentation to the hospital. In survivors, the duration of symptoms prior to presentation was 8.0 (3.8–11.3) days, and the length of hospitalization was 17.5 (13.8–21.3) days. Most patients (32/35) had blood cultures drawn within 24 hours of presentation and confirmed as *B. pseudomallei* 3.5 (2.0–5.0) days after admission. Survivors received positive results 4.5 (3.0–5.3) days after admission. Nonsurvivors died or were discharged to hospice within a median of 2.5 days from admission, but cultures did not return results until at least 3 days from admission (deaths: 2.0 (1–2.8) versus 3.5 (2.0–6.8), *P* = 0.0117; discharges to hospice: 2.0 (1.0–4.0) versus 3.0 (3.0–5.0), *P* = 0.0430) (Supplemental Table 2). For patients with complete drug administration logs (31/35), approximately 40% received appropriate antibiotics over the course of hospitalization (13/31). Fifteen patients died or were discharged to hospice without receiving appropriate antibiotic therapy, including two who received culture results before death or discharge. Patients who did not receive appropriate antibiotics had significantly higher odds of death than those who did (odds ration [OR]: 27.5, 95% CI: 4.1–148.2, *P* = .0003). When patients who were discharged to hospice were excluded, the odds of death remained significantly higher for those who did not receive appropriate antibiotics (OR: 14.67, 95% CI: 1.9–84.1, *P* = .0111).

## DISCUSSION

*Burkholderia pseudomallei* is a pathogen of increasing global relevance as suitable environments and at-risk populations grow. In this cohort, we demonstrated that administration of appropriate antibiotics was strongly associated with survival of patients with bacteremic melioidosis. Developing rapid diagnostics, providing access to first-line therapies, and honing empiric treatment algorithms are therefore critical to reducing mortality associated with the disease.

Rapid diagnostics can help ensure that appropriate antibiotics are administered quickly. Recent efforts to standardize and optimize diagnostic testing have been followed by increases in culture-confirmed melioidosis cases in Cambodia, but unavailability and inefficient use of laboratory resources (e.g., Ashdown selective media) have impeded widespread efforts, resulting in nonsystematic sampling of disease and incomplete surveillance on a national level.^8^

Cambodian microbiology laboratories depend primarily on blood cultures with basic biochemical panels (occasionally via bioMérieux API 20NE strips; Marcy-l′Étoile, France), more frequently manual testing for taxonomic identification. Three-disc tests, in which isolates are cultured on plates with discs containing an antibiotic (here, gentamicin, colistin and amoxicillin-clavulanate), are used to determine susceptibility.^8^ These methods require at a minimum 48–72 hours to return actionable results; we and others have found that most deaths occur within 72 hours of admission.^7^^,^^10^ One promising serology-based point-of-care test provides results in 15 minutes, after which patients with positive results for *B. pseudomallei* could present directly to regional hospitals rather than first seeking care at less-equipped community health centers.^13^ Such community-based screening methods implemented in high-risk populations could quickly identify potential cases and accelerate access to gold standard diagnostic testing.^14^

Although early diagnosis is the cornerstone of timely therapy, access to appropriate antibiotics is also key to reducing disease morbidity and mortality. In this case series, we noted that some patients did not receive appropriate antibiotics even after receiving a culture diagnosis. Many Cambodian hospitals report a lack of first-line antibiotics, leading to suboptimal therapy with ceftriaxone, co-trimoxazole, or shortened courses of ceftazidime before de-escalation to “eradication” therapy.^8^^,^^11^ Prioritizing availability of meropenem, for instance, may bolster empiric treatment given that nearly one-third of all pathogens isolated from blood cultures at Takeo Hospital during the study period were resistant to third-generation cephalosporins.

Ninety-one percent of the cases described here were diabetic, among the largest proportions in published melioidosis case series. Although diabetes has been well-described as a risk factor for bacteremia, there may have been additional unappreciated predisposing risks in this population (e.g., occupational hazards of rice farming) not present in other country reports.^5^ Patient charts were considered in their entirety, and other conditions significantly associated with bacteremia were infrequent or absent (i.e., chronic lung and kidney disease, immunosuppression, and malignancy).^5^ Globally, 50% or more of melioidosis patients have diabetes, which increases the risk of developing melioidosis at least 12-fold.^4^ In Cambodia, an estimated 596,000 adults aged 20–79 years have diabetes.^15^ Of these, 56.1% remain undiagnosed, a rate higher than that in 90% of the countries in the western Pacific region.^15^ Providers caring for patients with diabetes in *B. pseudomallei–*endemic regions should maintain heightened awareness of their risk for infection and consider early initiation of and/or rapid escalation to ceftazidime or carbapenems in patients presenting with syndromes consistent with melioidosis. However, the myriad presentations of patients with both diabetes and melioidosis, as highlighted above, may challenge such empiric treatment algorithms. Ultimately, more data are needed from disease-endemic, low-resource areas with a high prevalence of melioidosis and uncontrolled diabetes to help understand potential synergies in diagnostic and management strategies of these two highly morbid diseases.

The study’s principal limitation is its narrow population of bacteremic patients, who compose up to 75% of melioidosis cases reported elsewhere.^4^^–^^6^ Including less-severe presentations is important to inform comprehensive treatment guidelines; however, these nonsevere cases are rarely recognized and captured in Cambodia. When considering our 54% likely case-fatality rate, it is important to note that bacteremia has been significantly associated with worse outcomes and mortality rates upwards of 75%.^6^^,^^7^^,^^9^^,^^10^ Given that the estimated global mortality rate is also 54%, the true rate for all melioidosis cases in Cambodia is likely lower.^3^ Furthermore, exclusion of the one-third of patients for whom charts were unavailable may have introduced unforeseen bias.

Seasonal differences were sought in date of admission, length of hospitalization, appropriate treatment, site of infection, and outcomes, among other variables, but showed no significant correlations. However, the 1.5-year study period may have been too limited to appreciate seasonality or variations in access to melioidosis therapy that might explain inconsistent treatment across confirmed cases. Because this study was retrospective, we were unable to work with prescriber and laboratory stakeholders to change practices during the study period. We hope that by sharing results now, we raise awareness of the disease and associated morbidity and inform empiric prescribing for appropriate target populations. Future studies capturing potential exposure events, barriers to care, and adherence to antimicrobial therapy will further contribute to optimization of melioidosis diagnosis and treatment in Cambodia.

## Supplemental Materials

10.4269/ajtmh.23-0369 Supplemental Materials
