# Supplementary material for: Case Report: Risk Factors Associated with Mortality in Adults with Burkholderia Pseudomallei Bacteremia: A Retrospective Case Series of Melioidosis in Cambodia
Source: Am J Trop Med Hyg. 2024 Jun 18;111(2):277–80. doi: 10.4269/ajtmh.23-0369 (PMC11310626; doi:10.4269/ajtmh.23-0369)
Supplement: Supplemental Materials [file tpmd230369.SD1.pdf]

Table S1. Patient demographics and antibiotic resistance testing results for all pathogens isolated from blood cultures at Takeo Provincial Referral Hospital microbiology laboratory during study period (January 1, 2021 – June 15, 2022).

Culture Group	n (%)	Male, n (%)	Age, mean (SD)	Antibiotic	Resistant, n (%)	Intermediate, n (%)	Sensitive, n (%)
Enterobacterales	82 (46)	34 (41)	51.1 (24.2)	Ceftriaxone *(3 not tested)*	31 (39)	0	48 (61)
				Ceftazidime *(12 not tested)*	21 (30)	3 (4)	46 (66)
				Ciprofloxacin *(2 not tested)*	46 (58)	10 (13)	24 (30)
				Meropenem *(12 not tested)*	1 (1)	1 (1)	68 (97)
Acinetobacter sp.	8 (4)	6 (75)	41.5 (24.8)	Meropenem *(6 not tested)*	0	0	2 (100)
Staphylococcus aureus	14 (8)	11 (79)	23.0 (25.5)	Cloxacillin	5 (36)	0	9 (64)
Streptococcus sp.	10 (6)	7 (70)	48.1 (20.0)	Penicillin *(2 not tested)*	1 (13)	6 (75)	1 (13)
Other	64 (36)	42 (66)	52.5 (17.2)	-	-	-	-

Table S2. Demographics, clinical features and outcomes of 35 patients with bacteremic melioidosis.

		All patients (n=35) n (%)	Improved (n=16) n (%)	Died (n=10) n (%)	Discharged to hospice (n=9) n (%)
Demographic	**Age, mean (SD)**	56.1 (13.3)	56.2 (15.4)	57.6 (14.3)	54.1 (8.6)
	**Gender**				
	**Male**	26 (74)	11 (69)	8 (80)	7 (78)
	**Female**	9 (26)	5 (31)	2 (20)	2 (22)
	**Diabetes***	32 (91)	16 (100)	7 (70)	9 (100)
	**Hypoglycemic use in diabetic patients (n=31)±**				
	**Oral and/or insulin**	25 (78)	15 (94)	4 (57)	6 (75)
	**None**	6 (19)	1 (6)	3 (43)	2 (25)

	**Chronic alcohol use***				
	**No**	27 (77)	13 (81)	8 (80)	6 (67)
	**Yes**	8 (23)	3 (19)	2 (20)	3 (33)
	**First treated at ancillary facility**				
	**No**	24 (69)	13 (81)	5 (50)	6 (67)
	**Yes**	11 (31)	3 (19)	5 (50)	3 (33)
Clinical presentation	**Days of symptom duration before admission, median [IQR]**	4.0 [2.0-10.0]	8.0 [3.8-11.3]	3.5 [2.0-10.0]	2.0 [1.0-3.0]
	**Primary site of infection**				
	**Respiratory**	24 (69)	9 (56)	7 (70)	8 (89)
	**Skin/soft tissue**	5 (14)	2 (13)	3 (30)	0
	**Intraabdominal**	5 (14)	5 (31)	0	0
	**Urinary**	1 (3)	0	0	1 (11)
Clinical course	**Days hospitalized, median [IQR]**	4.0 [2.0-16.0]	17.5 [13.8-21.3]	2.0 [1.0-2.8]	2.0 [1.0-4.0]
	**Days from admission to positive culture, median [IQR]**	4.0 [2.5-6.0]	4.5 [3.0-5.3]	3.5 [2.0-6.8]	3.0 [3.0-5.0]
	**Appropriate antibiotics received†‡**				
	**Ever**	13 (37)	11 (69)	2 (20)	0
	**Never**	18 (51)	3 (19)	8 (80)	7 (78)

*Comorbid conditions were obtained from physician documentation

±Oral hypoglycemic was typically metformin, but 4 patients received glyburide alone or in combination with metformin; hypoglycemic data was incomplete for 1 patient (discharged to hospice) and excluded for 3 non-diabetic patients (all died)

†For acute treatment, appropriate antibiotics included ceftazidime and carbapenems

‡Medication data was incomplete for 4 patients (2 improved, 2 discharged to hospice)

Statistically significant ($p<0.05$) differences (quantitative data) and odds ratios (qualitative data) are shown in **boldface**
